# Influence of Financialization of Heavily Polluting Enterprises on Technological Innovation under the Background of Environmental Pollution Control

**DOI:** 10.3390/ijerph182413330

**Published:** 2021-12-17

**Authors:** Yingying Zhou, Yuehan Du, Fengyi Lei, Ziru Su, Yifei Feng, Jie Li

**Affiliations:** School of Economics and Management, China University of Mining and Technology, Xuzhou 221116, China; duyuehan@cumt.edu.cn (Y.D.); leifengyi@cumt.edu.cn (F.L.); suziru@cumt.edu.cn (Z.S.); fengyifei@cumt.edu.cn (Y.F.); lijiecumt@cumt.edu.cn (J.L.)

**Keywords:** heavily polluting enterprises, financialization, technological innovation, environmental pollution

## Abstract

In the wake of the acceleration of China’s industrialization and rapid economic growth, environmental pollution has also attracted great attention. The technological innovation of heavily polluting enterprises is conducive to reducing pollution emissions and promoting environmental health. The financial investment tendency and behavior of real enterprises have a significant impact on the technological innovation decision-making of enterprises. A panel model is used in this paper in order to empirically test the impact of financialization of Chinese heavily polluting enterprises on technological innovation based on the data of Listed Companies in Chinese heavily polluting industries from 2008 to 2019. The + results show that the financialization of heavily polluting enterprises has a significant crowding out effect on technological innovation. After introducing arbitrage motivation as the regulating variable, further research finds that arbitrage motivation weakens the inhibitory effect of enterprise financialization on technological innovation, that is, the stronger the arbitrage motivation, the smaller the negative effect of financialization on enterprise technological innovation, which weakens this crowding out effect. Finally, the listed enterprises in heavily polluting industries are divided into state-owned enterprises and non-state-owned enterprises according to their corporate attributes. Compared with state-owned enterprises, the financialization of non-state-owned enterprises has a greater squeeze out of technological innovation; and arbitrage motivation has a more significant regulatory effect on the impact of enterprise financialization on technological innovation.

## 1. Introduction

With the further development of ecological civilization, the 19th National Congress of the Communist Party of China put forward a new concept on environmental issues: “Optimize the ecological security barrier system, build ecological corridors and biodiversity protection networks, improve the quality and stability of the ecosystem, firmly establish the concept of socialist ecological civilization, and promote the formation of a new pattern of harmonious development of man and nature.” However, along with China’s accelerated industrialization process and rapid economic growth, environmental pollution has also received great attention. Currently, China’s economy is in a critical period of transformation, and is facing new challenges and opportunities. As the foundation of economic development, the real economy should be led by science and technology innovation to realize the transformation and upgrading of the real economy. As the financialization of China’s real economy has deepened in recent years, real enterprises, as the main force of technological innovation, tend to be detached from reality. At present, the financialization at the enterprise level is mainly reflected in the fact that most entity enterprises put a large amount of capital into financial activities such as stock investment and bank wealth management. In addition, a large amount of industrial capital flows to the field of investment real estate, and the phenomenon of “idling”, in which funds are away from the real economy in the virtual economy, is widespread. However, while there are risks in the financialization of enterprises, there are also certain opportunities. Due to the excessive profits in the financial market, corporate financial investment activities can increase the financing capacity of enterprises, and financial investment activities can also create sources of funds for technological innovation projects, and can also control the leverage ratio and the scale of financial investment within a reasonable range to achieve a reasonable and effective operation.

From the source of pollution, heavily polluting enterprises are the main body of responsibility for ecological environment pollution. In today’s world, ecological environment problems are mainly complicated and diversified, and there is a difficult situation of point-surface compounding, multi-source coexistence and multi-type overlay. Environmental pollution has become one of the problems that must be solved in the process of economic development in all countries of the world, and it is beneficial to promote the sustainable development of the global economy to deal with the relationship between environment and economy. Among them, technological innovation has become an indispensable and important link in the process of combating environmental pollution and improving environmental quality. In fact, in the initial stage of technological innovation, the use of resources and energy consumption caused by the expansion of economic scale and the development of industrial technology are the keys to environmental problems. However, Han Jian and Sheng Peihong [1], Huang Juan and Wang Mingjin [2], Ren Yayun and Zhang Guanglai [3] and other scholars believed that in the mature stage of technological innovation, continuous optimization of green technology innovation can develop clean energy, reduce the use of non-renewable energy, improve resource efficiency and productivity, thereby helping to improve the environment. Under the background of pollution control, technological innovation of heavily polluting enterprises is conducive to reducing pollutant emissions and promoting environmental improvement. At the same time, the gradual development of enterprise financialization has brought greater uncertainty to the development of real economy. Therefore, it is of great significance to study the direction and intensity of the influence of the financialization of heavily polluting enterprises on technological innovation. This is of great significance for heavily polluting enterprises in the critical stage of transformation and upgrading to achieve high-quality development and prevent excessive financialization of real enterprises. This paper takes the financialization of heavily polluting enterprises as the entry point, provides micro-evidence for the process of financialization of real economy, and enriches the existing research content.

## 2. Literature Review

As China’s economy enters a new era, innovation is the key to achieving stable transformation. The Outline of the 14th Five-Year Plan for National Economic and Social Development of the People’s Republic of China emphasizes the need to adhere to innovation-driven development and to comprehensively shape new advantages in development. Facing the challenges of the financial environment, the ability of enterprises to maintain sustained innovation is an important issue related to whether China’s economy can cross the middle-income trap. By combing through the relevant literature, we can find that studies on the impact of financialization of non-financial firms on firms’ technological innovation have not reached a uniform conclusion. According to their research findings, they can be broadly classified into three categories. First, the financialization of enterprises has played a “reservoir” effect on enterprise technology innovation. The second is the crowding-out effect of enterprise financialization on enterprise technology innovation. Third, the impact of enterprise financialization on enterprise technology innovation is not a simple promotion or inhibition relationship, but shows different characteristics under the influence of many factors.

Some researchers argue that the financialization of firms facilitates the alleviation of financing constraints, which improves their technological innovation, research and development capabilities. Kim et al. [4] and Almeida et al. [5] believed that firms with external financing constraints can maximize value by investing in liquid financial assets, which copes with cash flow shortages and ultimately promote innovative investments in the firm. Han and Qiu [6] believed that companies can invest funds in the allocation of financial assets to achieve the purpose of hedging and smoothing cash flow. Even in the case of a shortage of funds, the sale of more liquid financial assets can mitigate the financing constraints of a company, which increases its investment in innovation. Ju Xiaosheng et al. [7] showed that companies have lower adjustment costs and more liquid monetary funds and trading financial assets, which help reduce the risk of cash flow shortages and ease external financing constraints. In turn, it can promote the enterprise’s investment in technological innovation. Consider from the perspective of the long payback period and output uncertainty of technology R&D investment, HALL [8], Tadesse [9], Palley [10], Lazonick [11], Kliman and Williams [12], and Yang Zheng [13] all believed that investment in technology R&D requires sufficient or even excess capital investment. Investment in financial assets has the characteristics of a short payback period and high return, and the capital flow it obtains can make up for the shortage of funds for production and operation of enterprises. Therefore, the increase in the degree of financialization is conducive to improving the ability of R&D and innovation. Arizala et al. [14] and Gehringer [15] also proved that the increase in short-term investment returns will improve the efficiency and ability of firms to raise capital, which will alleviate the financing constraints of firms and ultimately increase their incentive to invest in technological innovation. Based on the channels of financial assets and liabilities, Sun Ping [16] believed that financial liability channel has a significant pull effect on corporate innovation, while the financial asset channel has no significant effect on corporate innovation. However, there is a significant crowding-out effect of corporate real estate investment on the scale of corporate innovation. Some scholars also classified the attributes of enterprises when studying the impact of enterprise financialization on enterprise technological innovation. Xu Shan, Liu Duchi [17], and Cui Guo [18] all believed that the impact of financialization on technological innovation of non-financial enterprises in China at this stage is mainly manifested as the “pull effect”. The “pull effect” of financialization on technological innovation of non-state enterprises is also higher than that of state-owned enterprises in terms of enterprise attributes.

Some researchers believe that enterprise financialization will have a crowding-out effect on enterprise technological innovation. Hu Yiming, Wang Xueting et al. [19], Wang Hongjian, Li Mangmang, Tang Taijie [20], Epstein et al. [21], Seo et al. [22], Demir [23], Akkemik [24], Stockhammer [25], Milberg et al. [26], and Crotty [27] all believed that the profit-seeking behavior of corporate managers will invest more of their limited capital in finance and focus too much on short-term interests, thus neglecting the development of their main business. This will further reduce the space for companies to invest in R&D and innovation. Scholars such as Ni Zhiliang and Zhang Kaizhi [28] believed that financialization significantly inhibits firms’ ability to innovate. Additionally, further research shows that the inhibitory effect of financialization on innovation is more pronounced for firms with severe financing constraints compared to those with relaxed financing constraints. Orhangazi [29] took American non-financial companies as the research object and found that financialization has a “crowding out effect” on industrial investment. In addition, Tori and Onaran [30] also reached similar conclusions on the financialization of companies in Turkey, Argentina, and Mexico, as well as on the financialization of non-financial companies in the UK. Based on the premise of limited enterprise resources, Xie Jiazhi et al. [31] found that excessive financialization of manufacturing enterprises inhibited technological innovation capabilities, while government control further magnified the negative impact of financialization on innovation. Having distinguished between long-term and short-term financial assets, Xu Gang and Zhu Weidong [32] found there was a negative relationship between corporate financialization and R&D investment intensity. Further study found that the inhibitory effect of long-term investment financialization on R&D investment is more significant than that of short-term speculative financialization on R&D investment. Ya Kun et al. [33], Huang Dayu and Xie Huobao et al. [34], Shi Xuezhi and Yang Zhen [35], and Yu Desheng and Li Xing [36] empirically concluded that excessive financial asset allocation will have a crowding out effect on enterprise innovation input. Additionally, economic policy uncertainty will aggravate this crowding out effect. Some scholars believed that the excessive allocation of financial assets by enterprises will have a crowding out effect on fixed investment and innovation investment. For example, Davis [37] found that shareholder value orientation affected firms’ management objectives, which in turn increases corporate investment in financial assets and ultimately discourages corporate investment in fixed assets and technological innovation. This effect is more pronounced in large non-financial firms. Some scholars have considered the impact of monetary policy while studying the financialization of firms. Zhang Chengsi and Zhang Butan [38] argued that the year-on-year increase in the share of financial income in net profits of non-financial firms were an important reason for the decline in technological innovation investment of real firms. Additionally, through empirical studies, it was found that financialization of the economy weakens the role of monetary policy in promoting the real economy, which ultimately weakened the incentive of enterprise technological innovation. This weakening effect increased with the deepening of the financialization of enterprises. Based on a multidimensional perspective, Zhao Liwei et al. [39] classified the financial assets of high-tech enterprises and found that financialization has a significant inhibitory effect on the technological innovation of high-tech enterprises. Additionally, it is mainly manifested in the financial assets of trading financial assets and investment real estate. Meanwhile, the inhibitory effect of financialization on technological innovation is more obvious in private enterprises and high profit enterprises. Jin Shengwu and He Shanshan [40], and Wang Jin [41] also explored from the perspective of property rights heterogeneity and found that corporate financialization has a significant crowding-out effect on technological innovation. In addition, financial assets show a significant crowding-out effect in state-owned enterprises. Zhang Yan et al. [42] and Guo Liting and Zhao Sutong [43] demonstrated through an empirical study that financialization of manufacturing firms has an overall inhibitory effect on innovation investment. Gu Haifeng and Zhang Huanhuan [44], Zhang Rui [45], Wang Yue [46], Xu Biao [47], Cheng Liwei and Xu Biao [48], Xiao Zhongyi et al. [49], and Duan Junshan and Zhuang Xudong [50] concluded from an empirical study that corporate financialization has a “crowding out” effect that inhibits continuous innovation of non-financial listed companies, with different effects on companies with different attributes.

Some scholars argue that the impact of firm financialization on technological innovation is not a simple facilitative or inhibitory relationship. Guo Liting [51] studied the investment decision process of manufacturing firms by constructing an asymmetric evolutionary game model. After empirical testing, it was found that the financialization of enterprises would have a “crowding-out effect” on their innovation investment, but this “crowding-out effect” would gradually evolve into a “water storage effect” with the improvement of business performance and the alleviation of financing constraints. According to Wang Hongjian et al. [52], the degree of financialization of enterprises shows a “positive U-shaped” relationship with their technological innovation investment. When the degree of financialization is less than 23%, it shows a crowding out effect on technological innovation, and gradually shows a pull effect after reaching 23%. Additionally, after considering the holding time of financial assets, Liu Guanchun [53] found that corporate financialization would have a significant crowding-out effect on current corporate technological innovation investment, but would have a boosting effect on the future technological innovation activities of enterprises. Wan Xuxian et al. [54] found that the holding of short-term financial assets by enterprises has no significant effect on dual innovation, while the holding of long-term financial assets will have a certain crowding-out effect on innovation. Further research found that equity incentives of SOEs can weaken the crowding-out effect of long-term financial asset allocation on dual innovation. Pan Haiying and Wang Chunfeng [55] found that the effect of financialization on innovation investment has a threshold effect. In other words, there is a reasonable range of fluctuations in the level of financialization of firms. When using technological innovation as a mediating variable, Chen Chiping and Kong Lixia [56] argued that corporate financialization leads to a decline in total factor productivity by “crowding out” technological innovation. Liu Bingrong [57] argued that corporate financialization has a significant inhibitory effect on innovation inputs and outputs of listed Chinese manufacturing firms, and has an “inverted U-shaped” effect on corporate innovation efficiency. Wang Shaohua [58] and other scholars also argue that corporate financialization has an “inverted U-shaped” effect on technological innovation. The study of Sun Zhihong and Zhou Ting et al. [59] showed that there is an unbalanced relationship between the effects of corporate financialization on the efficiency of capital allocation of real firms, and the proportion of innovation input of real firms has a moderating effect on the relationship between the two.

Most previous studies focused on the impact of enterprise financialization on enterprise technological innovation, and did not conduct separate studies based on the nature of the enterprise. Additionally, this article intends to expand the research from the following aspects based on the previous research experience: (1) When conducting empirical research on the impact of enterprise financialization on enterprise technological innovation, add arbitrage motivation as a moderating variable. (2) As the “Outline of the Fourteenth Five-Year Plan for the National Economic and Social Development of the People’s Republic of China” emphasizes the need to enhance the awareness of ecological and environmental protection in the whole society, and in-depth fight for pollution prevention and control. Therefore, heavily polluting enterprises will also become the focal point of attention of the whole society. This article sets the scope of enterprises in the heavily polluting industries, focusing on the impact of the financialization of heavily polluting enterprises on the technological innovation of enterprises. (3) Divide the sample into state-owned enterprises and non-state-owned enterprises to study separately, focusing on their differences.

## 3. Theoretical Analysis and Research Hypothesis

### 3.1. Theoretical Analysis of Enterprise Financialization and Technological Innovation

Milberg and Winkler [60] defined the financialization of the non-financial sectors as production-oriented enterprises that began to participate more in financial investment. More of the income obtained through financial investment is invested in the purchase of financial assets, and less in industrial investment. Cai Mingrong and Ren Shichi [61] defined enterprise financialization from two different aspects: results and behaviors. From a behavioral perspective, enterprise financialization means that most of the enterprise funds are used for investment rather than traditional production and operation activities. From the perspective of results, the financialization of enterprises is the main source of enterprise profits. The main source of enterprise profits has begun to change from financial investment activities, and the main business income as the main source of profits has been weakened. The capital cycle after enterprise financialization replaces the original capital cycle process, which is mainly manifested as: financial capital is gradually transformed into industrial capital; financial capital participates in the cycle of industrial capital through the borrowing behavior of economic entities in the capital market.

In *China’s Decisions on Strengthening Technological Innovation, Developing High* *Technology, and Realizing Industrialization*, technological innovation is defined as: technological innovation is a creative activity for enterprises to realize market value. Enterprises improve their existing products, production methods and operating modes by adopting new processes and new concepts, thereby improving the quality of their products and services, and ultimately expanding their market share by virtue of their advantages of product and service. However, uncertain sources of financing and high costs restrict the technological innovation activities of enterprises. In addition, the input cost for technological innovation is higher than the social cost, and the benefits obtained are lower than the social benefits. Therefore, many companies prefer to invest a higher rate of return on financial items in the case of limited funds.

### 3.2. Research Hypothesis

Through combing the relevant literature, it can be found that the research on the impact of non-financial enterprise financialization on technological innovation of enterprises has not reached a unified conclusion. According to the results of the research, it can be broadly divided into three categories. First, the financialization of enterprises has played a “water reservoir” effect on enterprise technological innovation. Second, the crowding-out effect of enterprise financialization on enterprise technology innovation. Third, the impact of enterprise financialization on enterprise technology innovation is not a simple promotion or inhibition, but shows different characteristics under the influence of a variety of factors. Xu Shan, Liu Dechi [17] and Cui Guo [18] all agreed that the impact of China’s financialization on the technological innovation of non-financial enterprises at this stage is mainly reflected in the “pull effect”. However, through theoretical analysis, this paper holds that today, with such a complex international economic situation, the heavily polluting industries are facing great pressure and greater uncertainty. When enterprises in the normal course of commodity business cannot get the expected return on investment, they will turn their attention to investments in financial assets with high returns and relatively short payback periods. In addition, technological innovation activities themselves have a long time, uncertain results and other high risks. In order to ensure the sustainability of enterprise innovation activities, heavily polluting enterprises must have sufficient retained earnings. However, in the case of limited capital, enterprises will inevitably give priority to the financial market, which leads to a significant reduction in investment in technological innovation activities. Demir [23] also proposed that enterprise managers give up long-term production targets for the sake of maximizing shareholder benefits, which aggravates short-term enterprise behavior, and affects or even interrupts investment in technological innovation. Therefore, Hypothesis 1 of this paper is proposed.

**Hypothesis** **1.** *The financialization of heavily polluting enterprises exerts a “crowding effect” on technological innovation of enterprises, and there is a negative correlation between the two.*

Market arbitrage is an activity in which entities put funds into financial markets to obtain excess returns. Some scholars have pointed out that due to limited enterprise capital, enterprise managers who invest funds into the financial market for market arbitrage motivations will inevitably crowd out the investment in enterprise technological innovation, thereby weakening the level of technological innovation [62]. Therefore, based on market arbitrage motivations, when companies invest in financial assets solely for arbitrage motivations, they will increase the scale of financial asset allocation by reducing R&D investment in technological innovation, thereby obtaining excess returns. The negative correlation between the two is more significant. Therefore, Hypothesis 2 of this paper is proposed.

**Hypothesis** **2.** *When the market arbitrage motivation of heavily polluting enterprises is stronger, the negative correlation between the degree of financialization and technological innovation capability is more significant.*

Since endogenous financing has excellent properties such as lowing financing costs, will not dilute the original shareholders’ equity, and reduce shareholders’ tax costs, enterprises will give priority to endogenous financing when considering financing channels. The net cash flow from operating activities is an important source of endogenous financing. When an enterprise faces a serious shortage of cash flow, it is more likely to invest the limited resources of its internal balance into financial investment with short payback period, rather than into technological innovation with a long cycle and uncertain returns. In this way, the normal production and operation activities are guaranteed. Therefore, the crowding out effect is more significant at this time [28]. When faced with ample cash flow, enterprise financial behavior may not only not squeeze out investment in technological innovation, but may also play a reservoir effect, thereby improving the level of technological innovation. Therefore, this paper proposes Hypothesis 3.

**Hypothesis** **3.** *Compared with heavily polluting enterprises with abundant cash flow, the more serious the cash flow shortage faced by the enterprise, the higher the possibility of arbitrage-driven financial assets, which has a more significant negative effect on technological innovation.*

There are significant differences in the degree of financialization between state-owned enterprises and non-state-owned enterprises in China [17,18]. Therefore, under this premise, the reservoir effect and the crowding out effect should be different. On the one hand, state-owned enterprises have a natural connection with the government. Compared with non-state-owned enterprises, state-owned enterprises are more likely to obtain government financial support and loans from financial institutions, so non-state-owned enterprises are more likely to choose financial assets with short payback periods and high yields because of limited funds; instead of choosing technological innovation activities. However, on the other hand, according to the principal-agent theory, due to the special and complex principal-agent chain of state-owned enterprises, the agency problem is more serious than that of non-state-owned enterprises, which makes state-owned enterprise managers pay more attention to short-term gains. State-owned enterprise managers are more inclined to give priority to financial investment projects that can improve financial performance in the short term, and give up long-term investment activities such as technological innovation activities. Therefore, this article proposes Hypotheses 4 and 5.

**Hypothesis** **4.** *The financialization of heavily polluting enterprises presents differences in enterprise attributes for technological innovation.*

**Hypothesis** **5.** *Arbitrage motivations show differences in enterprise attributes to the extent that the financialization of heavily polluting enterprises affects technological innovation of enterprise.*

## 4. Empirical Analysis

This paper mainly studies the impact of the financialization of heavily polluting enterprises on technological innovation, in the empirical analysis, that the years and industries will affect the level of technological innovation is taken into account. For example, first, the technological innovation ability and level changes over time, the technological innovation development in the past is slow, but is faster now; second, companies in different industries also have great differences in their technological innovation ability. Therefore, in this paper, a two-way fixed effect model that controls both year effect and industry effect is chosen, rather than a random effect model.

Learn from the previous research literature on financialization and technological innovation. This chapter establishes a quantitative model, selects listed companies in heavily polluting enterprises as research samples, and uses a two-way fixed effect model to empirically study the impact of financialization of listed companies in heavily polluting industries on technological innovation.

### 4.1. Index Selection, Model Setting and Sample Data Selection

(1) Quantification of enterprise technological innovation (ETI)

Xie Jiazhi [31], Zhao Liwei [39], and Guo Liting [43] all used the ratio of intangible assets to total assets to express the level of technological innovation. This article uses the ratio of intangible assets to total assets to quantify enterprise technological innovation indicators.

(2) Quantification of enterprise financialization (EF)

For heavily polluting entities, the proportion of financial assets in all assets can better reflect the behavior of heavily polluting enterprises in financial investment outside of normal business activities. Drawing on the domestic research of Song Jun [63], financial assets include trading financial assets, investment real estate, long-term equity investment, entrusted wealth management and trust products. Among them, trading financial assets include trading financial assets, derivative financial assets, net short-term investment, net available-for-sale financial assets, net held-to-maturity investment, net long-term debt investment, etc.

(3) Moderating variable

Introduce arbitrage motivation (AM) as a moderating variable. This article uses investment income/net profit to quantify enterprise market arbitrage motivation.

(4) Control variable

Cash flow (CF). This article uses the percentage of net cash flow from operating activities in total assets to quantify enterprise cash flow.

Debt-to-asset ratio (DAR). The level of technological innovation of enterprises is related to financing channels. The debt-to-asset ratio is the ratio of enterprise liabilities to assets. It reflects the external financing channels of enterprises. This article uses total liabilities/total assets to quantify.

Enterprise size (size). The size of the enterprise affects the financing constraints of the enterprise, and enterprise scale is represented by Ln (total assets).

Ownership concentration (OC). According to the theory of enterprise governance, the capital structure of an enterprise will have an impact on enterprise financialization or technological innovation. The degree of ownership concentration indicates the degree of dispersion or concentration of the enterprise’s equity, and the size of the shareholder’s right to speak in the enterprise. This indicator is also used to measure the internal stability of the enterprise. This article uses the proportion of the top ten shareholders to measure the degree of ownership concentration.

Growth rate of operating revenue (grow), Return On Total Assets (ROA), Capital Intensity (CI). The operating conditions and profitability of the enterprise will also have an impact on the technological innovation of the enterprise. The improvement in operating conditions and the increase in profitability will increase the enterprise’s disposable funds, which will have a certain impact on R&D expenditures. This article uses the ratio of fixed assets to total assets to measure capital intensity.

Time effect (ξ), industry effect (μ). With changes in the macro environment and policies, the level of technological innovation may vary greatly from year to year, so the time effect is introduced. If the enterprise is in the year, the value is 1; otherwise, it is 0. According to the “Guidelines for Environmental Information Disclosure of Listed Companies” (draft for comments) published by the Ministry of Environmental Protection on 14 September 2010, the heavily polluting industries are divided into 14 sub-sectors to set dummy variables. If the enterprise belongs to this industry, the value is 1, otherwise it is 0. See Table 1 for details.

Cash flow will directly affect the enterprise’s ability to invest funds in technological innovation research and development. The debt-to-asset ratio can indicate the enterprise’s external financing channels. The scale of the enterprise affects the extent to which the enterprise is constrained in financing. Ownership concentration can measure the degree of stability within the enterprise, and the stability within the enterprise will also affect the enterprise’s willingness to invest in technology research and development. Growth rate of operating revenue, total asset net interest rate, and capital intensity all affect the enterprise’s disposable funds, thereby affecting the enterprise’s willingness to invest in research and development. In order to verify the hypothesis, this article adds these variables to the model as control variables. Construct the following empirical models, Equations (1) and (2), in order to verify the impact of enterprise financialization on technological innovation.

(1) $$ETI_{it}=\alpha_{0}+\alpha_{1}EF_{it}+\alpha_{2}DAR_{it}+\alpha_{3}size_{it}+\alpha_{4}OC_{it}+\alpha_{5}grow_{it}+\alpha_{6}CI_{it}+\alpha_{7}ROA_{it}+\xi_{t}+\mu_{i}+\varepsilon_{it}$$

(2) $$\begin{array}{ll} ETI_{it}= & \alpha_{0}+\alpha_{1}EF_{it}+\alpha_{2}AM_{it}+\alpha_{3}EF_{it}*AM_{it}\;+\alpha_{4}DAR_{it}\;+\alpha_{5}size_{it}+\alpha_{6}OC_{it}+\alpha_{7}grow_{it}+\alpha_{8}CI_{it}\\ & +\alpha_{9}ROA_{it}\;+\xi_{t}+\mu_{i}+\varepsilon_{it} \end{array}$$

where ${}_{i}$ represents the industry and ${}_{t}$ represents the time. $\xi_{t}$ represents the time effect, and $\mu_{i}$ represents the industry fixed effect.

According to the “Guidelines for Environmental Information Disclosure of Listed Companies” (Draft for Comment) published by the Ministry of Environmental Protection on 14 September 2010, 196 listed companies in heavily polluting industries were selected by using wind database. The time period is 2008–2019. After selecting the data, the data was processed as follows: One is to eliminate ST companies; the second is to eliminate companies with incomplete, missing or abnormal financial data; the third is to exclude companies that have no financial assets or have not disclosed R&D expenditures; and the fourth is to standardize data for other continuous variables except dummy variables. In the end, 2352 observations were obtained. This article uses stata15 as the data analysis software.

### 4.2. Descriptive Statistical Analysis of Variables

Table 2 reflects the descriptive statistical results of the main variables selected in this article. For listed companies in heavily polluting industries, the average value of technological innovation is 0.0552, and the average value is greater than zero, indicating that heavily polluting industries still attach importance to investment in innovation. However, the minimum value is 0.00000196 and the maximum value is 0.5780. This data also shows that the overall level of technological innovation of listed companies in Chinese heavily polluting industries is extremely uneven, and there are large differences between companies. The average degree of financialization of listed companies in the heavily polluting industries is 0.0382. This result is greater than zero, indicating that most of the heavily polluting enterprises in China have financialized behaviors. Some companies’ financial assets account for as much as 70% of their total assets, but some companies have no financial assets. This shows that the degree of financialization of enterprises in Chinese heavily polluting industries vary greatly among individuals. The average value of operating net cash flow is 0.0641 and the standard deviation is 0.0740. This shows that the overall cash flow of listed companies in the heavily polluting industries is not sufficient, and the liquidity situation between companies is quite different.

As can be seen from Table 2, heavily polluting enterprises themselves are willing to carry out technological innovation, but the overall level of technological innovation is not high and individual differences are large; it can also be seen that heavily polluting enterprises have the trend of enterprise financialization, but the individual differences are great.

### 4.3. Variable Correlation Test

Table 3 shows the correlation matrix between explanatory variables and explained variables. It can be seen from the table that there is a strong correlation between the explanatory variables and the explained variables, and most of the variables are significantly correlated at the 1% level. Pearson correlation coefficients are relatively low, mostly are below 0.5, and the correlation coefficient between enterprise size (SIZE) and ownership concentration (OC) reaches 0.5061, but these two controls measure enterprise capabilities from different dimensions. Therefore, they can be put into the same model. The correlation coefficient between technological innovation (ETI) and financialization (EF) is −0.099, and is a significant negative correlation at the 1% level, which initially verifies Hypothesis 1 that there is a negative correlation between technological innovation and enterprise financialization. The correlation coefficient between technological innovation (ETI) and cash flow (CF) is 0.063, and shows a significant positive correlation at the 1% level. This provides partial support for Hypothesis 3 that the net cash flow of operating activities has a positive impact on the technological innovation of enterprises.

### 4.4. Multicollinearity Test

This article uses variable tolerance (Tolerance) and variance expansion factor (VIF) to accurately judge the multicollinearity between independent variables. Table 4 shows the results of collinearity diagnosis. The tolerance of all variables is higher than 0.1, and the variance expansion factor is between 1.01 and 1.59, which is much lower than 10. This shows that there is no serious multicollinearity problem between variables, and further regression analysis can be performed.

### 4.5. Regression Model Analysis

#### 4.5.1. Full Sample Regression

Table 5 is a summary of the regression results based on Model 1. The empirical result (1a) is the result of regression using a fixed-effect model (controlling time effect and industry effect at the same time) after adding control variables. Additionally, (1b) is the univariate regression result of only the dependent variable (ETI) and independent variable (EF) (still controlling the time effect and industry effect at the same time).

In univariate regression and multiple regression, the regression coefficients of enterprise financialization (EF) to enterprise technological innovation (ETI) are −0.0839 and −0.1009, respectively, and both results show a significant negative correlation at the 1% level. This result clearly shows that, for heavily polluting enterprises, enterprise financialization inhibits enterprise technological innovation, that is, to verify that Hypothesis 1 is true. This is in line with the conclusion of Hu Yiming and other scholars [19,20] that the financialization of enterprises inhibits enterprise technological innovation. With the continuous and in-depth development of Chinese financial industry, more and more heavily polluting enterprises have begun to increase their capital investment in financial assets. An important cause of this phenomenon is that financial assets can obtain excess returns in a relatively short period of time, while the investment in technological innovation faces both a long R&D time and a situation where there is no benefit if it fails. In the case of limited funds for heavily polluting enterprises, most managers will invest their precious funds in the allocation of financial assets instead of investing in technological innovation. As a result, enterprise financialization will have a crowding-out effect on enterprise technological innovation.

From the perspective of enterprise financing sources, the coefficient of enterprise net operating cash flow (CF) to enterprise technological innovation (ETI) is 0.0514, and shows a significant positive correlation at the 5% level. From the coefficient, it can be seen that the net operating cash flow of an enterprise has played a significant role in promoting the technological innovation of the enterprise. That is, Hypothesis 3 holds. The net operating cash flow of an enterprise is an important part of the enterprise’s internal financing. When enterprise managers have sufficient internal funds, companies will consider investing funds in technological innovation for obtain longer-term profits, instead of just pursuing immediate benefits. The debt-to-asset ratio (DAR) has a coefficient of 0.0056, but its impact on enterprise technological innovation is not robust. However, it may be possible to conclude from the symbol that enterprise debt is conducive to technological innovation. It shows that when listed companies in heavily polluting industries receive external financing, they will spend more on technological innovation.

From the perspective of ownership concentration, the coefficient of ownership concentration (OC) for enterprise technological innovation is −0.0345, showing a significant negative correlation at the 1% level. This is the same as the conclusion drawn by a scholar named Guo Liting [51]. This result clearly shows that the higher the ownership concentration in a polluting enterprise, the more unfavorable the technological innovation of the enterprise. The high ownership concentration indicates that the decision-making power of the enterprise is only in the hands of minority shareholders, which is likely to be detrimental to the reasonable allocation of the enterprise’s limited resources. Moreover, senior managers are likely to damage the long-term development of the enterprise because of high short-term profits, so they choose to invest funds in the allocation of financial assets instead of technological innovation.

From the perspective of enterprise size, the coefficient of enterprise size (size) to enterprise technological innovation is −0.0407, and shows a significant negative correlation at the 10% level. It shows that the scale of the enterprise has restrained the investment in technological innovation. This seems to be completely contrary to the conclusions reached by scholars such as Ni Zhiliang and Zhang Kaizhi [28]. This also seems to be contrary to what is believed in daily life that “the larger the scale, the stronger the ability to innovate”, but this precisely illustrates the shortcomings of the future development of China’s current heavily polluting industries. The first reason is that large-scale companies are relatively advanced in technology, and technological innovation is facing difficulties in breakthroughs, which makes it impossible for companies to innovate. The second is that the larger the enterprise, the more likely it is to be satisfied with today’s technological level. They tend to give priority to current interests rather than long-term enterprise development, and thus are unwilling to invest in technological innovation. However, for relatively small and heavily polluting enterprises, technological innovation is the fundamental driving force for their progress. Therefore, smaller companies are more willing to invest funds in technological innovation and rely on technology to demonstrate their core competitiveness.

From the perspective of enterprise operating conditions, return on total assets (ROA) has a negative coefficient for enterprise technological innovation and is significant at the level of 1%. This shows that the stronger the profitability of an enterprise, the more restrained the technological innovation ability of the enterprise. This result shows that most of the managers of heavily polluting enterprises will choose financial investments with fast returns and high returns because they care about the profits on the current income statement, rather than investing in technological innovation. The better the profitability, the more likely it is to attract managers to invest funds in financial assets, thereby obtaining more excess returns. The growth rate of operating income (grow) is not significant for the coefficient of enterprise technological innovation. This shows that the increase in business income of an enterprise is not necessarily related to the ability of the enterprise’s innovative activities, and the increase in total revenue does not mean an increase in enterprise profits. The coefficient of capital intensity (CI) for enterprise technological innovation is negative, and is significantly negatively correlated at the 10% confidence level. This result shows that the higher the capital intensity of an enterprise, the lower the level of technological innovation of the enterprise. The high capital intensity of an enterprise indicates that the enterprise has more fixed assets and relatively less current assets. When an enterprise faces limited liquidity assets, the managers of heavily polluting enterprises will choose to invest funds in financial assets that can quickly return the enterprise, thereby creating a crowding-out effect on the enterprise’s technological innovation.

#### 4.5.2. The Moderating Effect of Arbitrage Motivation on Enterprise Financialization and Technological Innovation Capability

**Figure 1:**
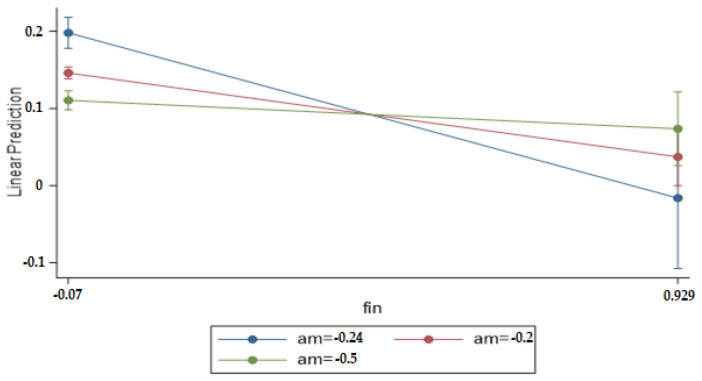
Diagram of the moderating effect of arbitrage motivation (adjusted predictions with 95% CIs).

The motives of high-level managers of heavily polluting enterprises for investment decisions will largely affect the flow of investment funds. The moderating effects of arbitrage motivations on the financialization of heavily polluting enterprises and their technological innovation show that: Firstly, the three groups of companies with different arbitrage motivations are not parallel, indicating that the arbitrage motivations of heavily polluting enterprises have a moderating effect on enterprise financialization and enterprise technological innovation. Secondly, the group with stronger arbitrage motivations is weaker than the group with weaker arbitrage motivations in negatively regulating the effect of enterprise financialization on enterprise technological innovation. In general, the arbitrage motivation of enterprise investment decision-making has a moderating effect on the impact of enterprise financialization on enterprise technological innovation. Figure 1 shows the moderating effect of arbitrage motivation.

In order to verify Hypotheses 2 and 3, the variable AM, which measures enterprise arbitrage motivation, is introduced in Model 2; and the interaction term EF*AM, which is the interaction term between financialization and market arbitrage motivation, is introduced to perform regression. Table 6 is a summary of the regression results based on Model 2. The empirical result (2a) is the regression result of the fixed-effect model (controlling the time effect and industry effect at the same time) after adding the control variables. Additionally, (2b) is the regression result without adding control variables (still controlling the time effect and industry effect at the same time).

Table 6 presents the test results of the moderating effect of arbitrage motivation. According to the results of (2a) and (2b), it can be concluded that after the interaction term (EF*AM) is added, the coefficients of the financialization of heavily polluting enterprises for enterprise technological innovation are −0.1535 and −0.0896, respectively, and both are significant at the level of 1%. This shows that this is consistent with the previous empirical results, and the financialization of heavily polluting enterprises is still a clear crowding-out effect on enterprise technological innovation. The coefficients of the interaction term between arbitrage motivation and enterprise financialization (EF*AM) are 0.25 and 0.1702, respectively, and both are significant at the 5% level. This result shows that the arbitrage motivation has a significant moderating effect on the financialization of heavily polluting enterprises and their technological innovation. The positive coefficient indicates that the arbitrage motivation has weakened the negative effect of enterprise financialization on enterprise technological innovation. That is, Hypothesis 2 does not hold. Although China’s current financialization of heavily polluting enterprises will have a crowding effect on enterprise technological innovation, arbitrage motivation weakens the negative impact of enterprise financialization on enterprise technological innovation. This may be due to the higher the arbitrage motivation of the heavily polluting enterprises, the more financially they can solve the problem of information asymmetry between the heavily polluting enterprises and financial institutions in more ways. In the end, the more conducive to enterprise financing and investment in technological innovation. The financial investment income obtained by heavily polluting enterprises based on arbitrage motivations enables heavily polluting enterprises that are trapped in financing constraints to obtain richer enterprise financing channels, expand the internal capital market, and alleviate the lack of innovation resources. These benefits make heavily polluting enterprises have more abundant funds to carry out technological research and development. After adding the arbitrage motivation, the coefficient of enterprise net operating cash flow (CF) to enterprise technological innovation (ETI) is 0.0501, and shows a significant positive correlation at the 5% level. It is consistent with the previous empirical results, that is, Hypothesis 3 holds.

#### 4.5.3. Based on the Difference between State-Owned and Non-State-Owned Listed Companies in Heavily Polluting Industries

State-owned enterprises and non-state-owned enterprises are quite different in terms of organizational structure, social resources, and development goals. Therefore, heavily polluting enterprises are divided into state-owned holding enterprises and non-state-holding enterprises according to their enterprise attributes. After being divided into two categories, regression analysis was performed, respectively. The regression results are presented in Table 7. In univariate regression and multiple regression, the coefficients of enterprise financialization of non-state-owned heavily polluting enterprises on enterprise technological innovation are −0.1708 and −0.2066, respectively, and both are significant at the 1% confidence level. The results show that the financialization of non-state-owned heavily polluting enterprises is still an obvious crowding-out effect on enterprise technological innovation. However, compared with non-state-owned enterprises, whether in univariate regression or multiple regression in state-owned enterprises, the regression results of enterprise financialization on enterprise technological innovation are not significant at the 10% confidence level. Therefore, Hypothesis 4 is established, that is, the financialization of heavily polluting enterprises presents differences in enterprise attributes for technological innovation.

In order to verify Hypothesis 5, the interaction term EF*AM of financialization and market arbitrage motivation are introduced at the same time, and the regression is carried out. (2c) and (2d), respectively, present the test results of the moderating effect of the arbitrage motivations of state-owned enterprises and non-state-owned enterprises. According to the results, after adding the interaction term (EF*AM), the coefficient of financialization of non-state-owned heavily polluting enterprises for enterprise technological innovation is −0.2614, which is significant at the level of 1%. This result is consistent with the previous empirical results, indicating that the financialization of non-state-owned heavily polluting enterprises is still a significant crowding-out effect on enterprise technological innovation. The coefficient of the interaction term between arbitrage motivation and enterprise financialization (EF*AM) is 0.2337, which is significant at the level of 10%. This result shows that the arbitrage motivation has a significant moderating effect on the financialization of non-state-owned heavily polluting enterprises. However, the coefficient of enterprise financialization of state-owned heavily polluting enterprises on enterprise technological innovation is not significant at the 10% confidence level. This shows that the moderating effect of arbitrage motivation does not exist. The above results verify the establishment of Hypothesis 5, that is, the degree of arbitrage motivations that affects the financialization of heavily polluting enterprises and the extent to which they affect technological innovations shows differences in enterprise attributes.

The above results show that the financialization of heavily polluting enterprises has a greater impact on non-state-owned enterprises’ technological innovation than that of state-controlled enterprises. The first reason is that compared with state-owned enterprises, non-state-owned enterprises have fewer social resources and their financing channels are relatively limited. In the case of limited enterprise funds, financial investment by non-state-owned enterprises has a more obvious effect of crowding-out R&D expenditure for technological innovation. The second is that senior managers of non-state-owned enterprises will pay more attention to individual final evaluation indicators. For their personal performance, they may choose short-term benefits with quick and high returns, thus giving up investment in technological innovation with a long payback period.

### 4.6. Robustness Test

In order to further verify the reliability of the empirical results of this paper, this paper will conduct the following reliability tests. In the above empirical research, this article mainly uses the fixed effects model, and then adopts the FGLS method to revalidate the empirical results. The regression results are presented in Table 8. The coefficient of the financialization of heavily polluting enterprises on enterprise technological innovation is −0.0588, showing a significant negative correlation at the 5% confidence level. The coefficient of the interaction term (EF*AM) between arbitrage motivation and enterprise financialization is 0.0749, showing a significant positive correlation at the level of 10%. The coefficient of net enterprise operating cash flow (CF) to enterprise technological innovation (ETI) is 0.0442, showing a significant positive correlation at the 5% level. From the perspective of the direction and significance of the influence coefficient of the core explanatory variables, it is consistent with the results of the previous empirical analysis. It further demonstrates that the financialization of heavily polluting enterprises will have a serious crowding-out effect on enterprise technological innovation. Arbitrage motives have a significant moderating effect on the financialization of heavily polluting enterprises and their technological innovation. The net operating cash flow of heavily polluting enterprises has played a significant role in promoting technological innovation of enterprises.

## 5. Conclusions

China, as a responsible developing country, is facing the pressure of environmental pollution, and heavily polluting enterprises need to achieve high-quality development in a more energy-saving and environmentally friendly way. It is crucial for China to find the path and countermeasures to achieve high-quality economic development to deal with environmental problems. Under the general trend of China’s economic financialization, heavily polluting enterprises have joined the trend of enterprise financialization in succession. We can find and solve the problem of low average level of technological innovation of heavily polluting enterprises by studying the impact of financialization of heavily polluting enterprises on technological innovation, which is conducive to further exploring the field of technological innovation to reduce environmental pollution. With the continuous improvement of the technical level of heavily polluting enterprises, the utilization of resources will be more reasonable and efficient. This will play an effective role in promoting heavily polluting enterprises to further reduce environmental pollution and reduce resource waste while maintaining economic benefits.

Technological innovation is one of the core drivers of enterprise development at present. With the rapid development of the Chinese market, enterprises need to constantly update equipment, technology, and research new products to improve their core competitiveness. Technological innovation investment needs to occupy a large amount of liquidity of enterprises themselves, and most enterprises have a long technology renewal cycle and a high risk of failure, so enterprise managers are often extra cautious in making technological innovation decisions. Therefore, business managers are often extra cautious when making technological innovation decisions. The financial market in our country has shown a trend of rapid development in recent years, and new financial derivatives are constantly appearing. This allows non-financial companies that are pursuing higher and faster investment returns to participate, and the trend of financialization of entity companies has emerged. In the context of China’s advocacy of green development, it is particularly meaningful to study the impact of the financialization of heavily polluting enterprises on technological innovation. This paper takes 196 heavily polluting enterprises listed on China’s A-shares as a research sample, and explores the impact of the financialization of China’s heavily polluting enterprises on technological innovation through theoretical analysis and empirical research. The specific conclusions are as follows:

The financialization of heavily polluting enterprises has inhibited the technological innovation of enterprises, and has a significant crowding-out effect on technological innovation investment. This finding is consistent with the majority of previous scholars [28,29,30,31] who concluded that the financialization of firms severely inhibits the level of technological innovation of firms. Additionally, this paper verifies that financialization of heavily polluting enterprises also inhibits firm technological innovation, starting from heavily polluting enterprises. This is also proved in the empirical research and in the process of reliability testing. This result can be explained from both the difficulties faced by domestic heavily polluting enterprises and the advantages of financial investment. First, China is currently vigorously advocating green development of the economy, and heavily polluting enterprises are facing difficult problems such as difficult transitions and declining enterprise profit margins. These problems have greatly weakened the enthusiasm for investment in technological innovation. Second, the rapid development of the financial industry, the continuous updating of financial investment methods, and the characteristics of fast returns and high returns attract managers of heavily polluting enterprises to join the capital market. Therefore, the main business was neglected, and the investment in technological innovation was inadequate.

The net cash flow of an enterprise’s operating activities is an important channel for the enterprise’s endogenous financing, which has a significant positive correlation with technological innovation. It shows that the net cash flow of the business activities of the enterprise has played a significant role in promoting the technological innovation of the enterprise. Increasing operating net cash flow is beneficial for companies. When the enterprise’s own circulating funds are sufficient, senior managers are still willing to invest funds in the research and development of technological innovation for the long-term development and competitiveness of the enterprise.

Arbitrage motivations have weakened the restraining effect of financialization on technological innovation. Introduce the interaction term of financialization and arbitrage motivation in the empirical model. The coefficient of enterprise financialization on enterprise technological innovation is still significantly negative, but the coefficient of the interaction term between financialization and arbitrage motivation is significantly positive. This shows that the stronger the arbitrage motivation is, the less negative effect of financialization on enterprise technological innovation will be, which weakens the crowding-out effect. The reason for this result is that based on arbitrage motivations, the income from financial investment can flow into technological innovation research and development activities, so as to alleviate the pressure caused by the shortage of research and development funds.

Compared with state-owned enterprises, the financialization of non-state-owned enterprises has a greater degree of crowding-out technological innovation; arbitrage motivations have a more significant moderating effect on enterprise financialization that affects enterprise technological innovation. Compared with non-state-owned enterprises, state-controlled enterprises are more deeply affected by national policies. The state currently strongly supports scientific and technological innovation and offers many preferential treatments including taxation in terms of technological research and development. Therefore, from the perspective of capital constraints, under the circumstance of limited enterprise funds, financial investment by non-state-owned holding companies has a more obvious crowding-out effect on technological innovation R&D expenditure. From the perspective of business managers, senior managers of non-state-owned enterprises will pay more attention to personal final evaluation indicators. For the sake of personal performance, they will choose financial investments with fast returns and high yields, thus giving up investment in technological innovation with a long payback period.

## Figures and Tables

**Table 1:** The meaning and calculation method of the main variables of the model.

Variable Type	Variable Meaning	Variable Code	Variable Definitions
Dependent variable	Enterprise Technological Innovation	ETI	ETI = Intangible assets/total assets
Independent variable	Enterprise Financialization	EF	EF = Financial assets/total assets
moderating variable	Arbitrage Motivation	AM	AM = Investment income/net profit
Control variable	Debt-to-asset Ratio	DAR	DAR = Total liabilities/total assets
	Enterprise Size	size	Size = Ln(Total assets)
	Ownership Concentration	OC	OC = Shareholding ratio of the top ten shareholders
	Return On Total Assets	ROA	ROA = Net profit/total assets
	Capital Intensity	CI	CI = Fixed assets/total assets at the end of the period
	Operating Income Growth Rate	grow	Grow = (Operating income for the current period − operating income for the previous period)/Operating income of the previous period
	Cash Flow	CF	CF = Net cash flow from operating activities/total assets
virtual variable	Time Effect	ξ	Set the time dummy variable, if the enterprise is in the year, the value is 1, otherwise it is 0.
	Industry Effect	μ	Set the industry dummy variable, if the enterprise is in the industry, the value is 1, otherwise it is 0.

**Table 2:** Descriptive statistics of main variables.

Variable	Mean	Standard Deviation	Min	Max
ETI	0.05521830	0.05652120	0.00000196	0.57801470
EF	0.03819390	0.07461390	0.00000000	0.70971660
DAR	0.47603130	0.20961520	0.00707990	2.02392200
size	22.86968000	1.49925200	19.09337000	28.63649000
OC	0.57008610	0.16308280	0.00000000	0.98590000
grow	0.20598610	1.48569700	−0.95704060	58.35673000
CI	0.29761580	0.16577220	0.00000000	0.82472470
ROA	0.04508320	0.07746950	−1.03751900	1.12612100
Cf	0.06414810	0.07404280	−0.25524930	0.43817810

**Table 3:** Correlation matrix of main relevant variables.

	ETI	EF	DAR	Size	Grow	CI	ROA	CF	OC
**ETI**	1								
**EF**	−0.0990 ***	1							
**DAR**	0.0367 *	−0.1323 ***	1						
**size**	−0.0066	−0.1488 ***	0.2871 ***	1					
**grow**	0.0143 ***	0.0456 **	−0.0406 **	0.0449 **	1				
**CI**	−0.0523 **	−0.2374 ***	0.2925 ***	0.3237 ***	−0.0263	1			
**ROA**	0.0812 ***	0.0271	−0.324 ***	−0.0128	0.1850 ***	−0.0883 ***	1		
**CF**	0.0633 ***	−0.1130 ***	−0.2018 ***	0.0999 ***	−0.0954 ***	0.1602 ***	0.4285 ***	1	
**OC**	−0.0296	−0.1949 ***	0.0252	0.5061 ***	0.0319	0.1810 ***	−0.0585 ***	0.0990 ***	1

Note: ***, ** and * are significant at 1%, 5% and 10% significance levels, respectively.

**Table 4:** Multicollinearity test results.

Variable	VIF	Tolerance
EF	1.12	0.893455
OC	1.42	0.703868
DAR	1.52	0.659189
size	1.55	0.644647
grow	1.01	0.988526
CI	1.32	0.756582
ROA	1.59	0.628976
CF	1.40	0.714559
Mean VIF	1.37	

**Table 5:** Full-sample regression results of the impact of financialization of listed companies in heavily polluting industries on technological innovation.

Explanatory Variables	(1a)	(1b)
EF	−0.1009 *** (−5.02)	−0.0839 *** (−4.32)
DAR	0.0056 (0.33)	
CF	0.0514 ** (2.43)	
size	−0.0407 * (−1.89)	
OC	−0.0345 ** (−2.24)	
grow	0.0281 (1.03)	
CI	−0.0255 * (−1.69)	
ROA	−0.083 *** (−2.69)	
constant	0.3944 *** (15.85)	
Time effect	control	control
Industry effect	control	control
F value	18.9700	24.5300
Prob(F-statistic)	0.0000	0.0000
adj-R^2^	0.1965	0.2002
obs	2352	2352

Note: () is *t*-test statistic; ***, ** and * are significant at 1%, 5% and 10% significance levels, respectively.

**Table 6:** Test results of the moderating effect of arbitrage motivations.

Explanatory Variables	(2a)	(2b)
EF	−0.1535 *** (−4.43)	−0.0896 *** (−4.49)
AM	−0.0868 ** (−2.07)	−0.0619 * (−1.81)
EF * AM	0.2500 ** (2.06)	0.1702 ** (2.03)
DAR	0.0075 (0.45)	
CF	0.0501 ** (2.37)	
size	−0.0404 * (−1.88)	
OC	−0.0338 ** (−2.20)	
grow	0.0280 (1.02)	
CI	−0.0258 * (−1.71)	
ROA	−0.0847 *** (−2.75)	
constant	0.4053 *** (15.96)	
Time effect	control	control
Industry effect	control	control
F value	18.0600	21.4700
Prob(F-statistic)	0.0000	0.0000
adj-R^2^	0.1979	0.1903
obs	2352	2352

Note: () is *t*-test statistic; ***, ** and * are significant at 1%, 5% and 10% significance levels, respectively.

**Table 7:** Regression results of listed companies in heavily polluting industries by enterprise attributes.

Explanatory Variables	State-Owned Holding			Non-State Holding		
	(1c)	(1d)	(2c)	(1e)	(1f)	(2d)
EF	−0.0188 (−0.64)	−0.0175 (−0.58)	−0.0233 (−0.75)	−0.1708 *** (−6.34)	−0.2066 *** (−7.26)	−0.2614 *** (−5.82)
AM			−0.0488 (1.32)			−0.1854 ** (−2.08)
EF*AM			0.1902 * (1.70)			0.2337 * (1.83)
CF		0.1050 *** (3.81)	0.1046 *** (3.78)		−0.026 (−0.80)	−0.0295 *** (−0.90)
DAR		0.0661 *** (3.11)	0.0688 *** (3.23)		−0.1065 *** (−3.82)	−0.1028 *** (−3.69)
size		−0.0407 (−1.45)	−0.0432 (−1.54)		−0.0119 (−0.28)	−0.0094 (−0.22)
OC		0.0064 (0.29)	0.0080 (0.36)		−0.0913 *** (−4.03)	−0.0885 *** (−3.90)
grow		−0.0445 (−1.24)	−0.0456 (−1.28)		0.0851 ** (2.00)	0.0824 * (1.93)
CI		−0.0477 *** (−2.63)	−0.0480 *** (−2.65)		0.0207 (0.75)	0.0173 (0.62)
ROA		−0.0321 (−0.75)	−0.0331 (−0.78)		−0.1447 *** (−3.20)	−0.1429 *** (−3.17)
constant		0.2913 *** (9.15)	0.2959 *** (9.17)		0.6195 *** (13.72)	0.6195 *** (13.72)
Time effect	control	control	control	control	control	control
Industry benefits	control	control	control	control	control	control
F value	19.4500	15.9300	15.0600	8.9300	8.5700	8.2400
Prob(F-statistic)	0.0000	0.0000	0.0000	0.0000	0.0000	0.0000
adj-R^2^	0.2206	0.2301	0.2308	0.1827	0.2161	0.2191
obs	1500	1500	1500	852	852	852

Note: () is *t*-test statistic; ***, ** and * are significant at 1%, 5% and 10% significance levels, respectively.

**Table 8:** Robustness test regression results.

Explanatory Variables	(2e)
EF	−0.0588 ** (−2.19)
AM	−0.0457 * (−1.94)
EF*AM	0.0749 * (1.78)
DAR	0.0782 *** (2.87)
CF	0.0442 ** (2.07)
size	−0.0406 (−1.00)
OC	−0.0331 ** (−2.21)
grow	−0.0129 (−0.52)
CI	0.0206 (1.49)
ROA	0.0444 ** (2.24)
Time effect	control
Industry effect	control
Prob > chi2	0.0000
R^2^	0.2656
obs	2352

Note: () is *t*-test statistic; ***, ** and * are significant at 1%, 5% and 10% significance levels, respectively.
